# Operation for Congenital Phymosis

**Published:** 1838-03-14

**Authors:** Thomas Harris


*Operation for congenital Phymosis. By* Thomas Harris, M. D.

This case was that of a lad, seventeen years of age. Dr. Harris cut off a quarter of an inch of the prepuce, and afterwards slit up the internal membrane with a bistoury. The inner and outer edges were then stitched together by several sutures, and a single dressing applied. Discharged, cured in four weeks.

